# The role of silver additives in gold-mediated C–H functionalisation

**DOI:** 10.3762/bjoc.7.102

**Published:** 2011-07-01

**Authors:** Scott R Patrick, Ine I F Boogaerts, Sylvain Gaillard, Alexandra M Z Slawin, Steven P Nolan

**Affiliations:** 1 EaStCHEM School of Chemistry, University of St Andrews, North Haugh, St Andrews, KY16 9ST, UK

**Keywords:** C–H functionalisation, gold catalyst, halide abstraction, *N*-heterocyclic carbene, silver salt, substrate activation

## Abstract

The role of silver additives is examined in the context of gold-mediated functionalisation of aromatic C–H bonds. Doubt is cast on the commonly cited route of halide abstraction from gold and evidence of substrate activation is given.

## Introduction

**Scheme 1:**
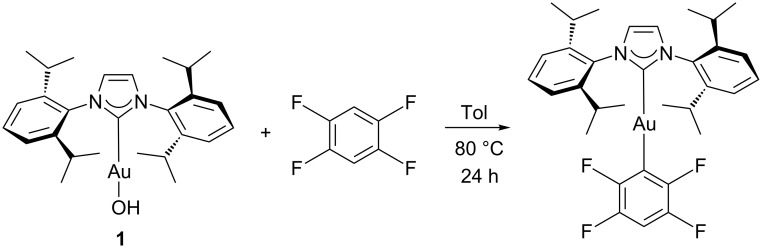
Silver-free C–H functionalisation using [Au(OH)(IPr)].

The use of gold in homogeneous catalysis is an area where fascinating advances have been realised in the last few years [1–4]. One of these discoveries has focused on the possibility of using gold in metalation reactions [5–8] directly leading to organogold complexes. The further use of organogold complexes bearing *N*-heterocyclic carbenes (NHC) [9–10] as supporting ligands has enabled the isolation of a “golden synthon”, [Au(OH)(IPr)] **1** (IPr = 1,3-bis(2,6-diisopropylphenyl)imidazol-2-ylidene), that is able to participate in metalation reactions with aromatic C–H bonds (Scheme 1) [11].

The reactivity profile of **1** appears linked to bond acidity, and was found to functionalise selectively the most electron-deficient C–H bond in a substrate. Unfortunately, the reactivity of **1** was limited to bonds with a p*K*_a_ value below 30.4 [12].

**Scheme 2:**
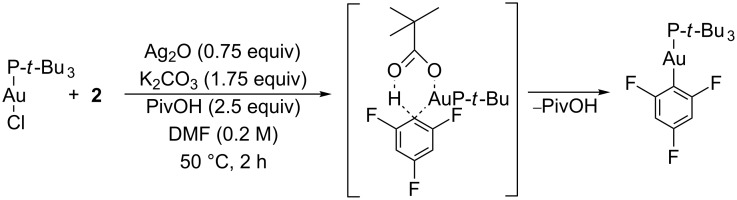
C–H functionalisation of **2** using gold-phosphine complexes and a silver additive.

Current gold research has shown that silver salts can not only improve reaction times and yields [13–14], but also allow the C–H functionalisation of previously unreactive substrates. Larrosa has recently disclosed a mild methodology for the Au(I)-mediated C–H functionalisation of 1,3,5-trifluorobenzene (**2**, p*K*_a DMSO_ 31.5 [15]) using a mixture of reagents and additives (Scheme 2) [16]. The observation of a high kinetic isotope effect is suggestive of a concerted metalation–deprotonation mechanism, as first suggested for Pd(II) complexes, in which a pivalate ligand behaves as a proton acceptor via a six-membered transition state [17]. However, addition via a transient Au(III) hydride would also be consistent with these observations.

## Results and Discussion

We became interested in understanding the mechanistic details of this transformation by identifying the role of the individual components. Reactions were performed between individual reagents and the formation of products was observed by ^1^H NMR spectroscopy. Silver(I) oxide and potassium carbonate both reacted with pivalic acid to form metal pivalates after stirring in toluene at 50 °C for 20 h. Potassium carbonate did not interact directly with the aryl substrate after stirring in DMF at 50 °C for 24 h. A stoichiometric reaction between Ag_2_O and the substrate displayed substitution of one of the protons. However, deuterium incorporation experiments were unsuccessful and mass spectrometry on the product was inconclusive.

The reaction depicted in Scheme 2 was performed using a neutral gold(I)-NHC complex. The reaction time was extended to 20 h in order to obtain optimal results. A reaction involving [AuCl(IPr)] proceeded with full conversion to product with all additives/reagents present. The removal of potassium carbonate from the reaction had no detrimental effect. Additionally, when it was the only additive used, no reaction took place. These results clearly eliminate the involvement of K_2_CO_3_ in direct deprotonation of the substrate [18]. Potassium carbonate reacts with pivalic acid to form KOPiv, leading to improved yields in the Larrosa methodology. However, this practice was not necessary in our work and the salt did not appear to have any other role in the mechanism. Further reactions did not utilise K_2_CO_3_.

**Figure 1:**
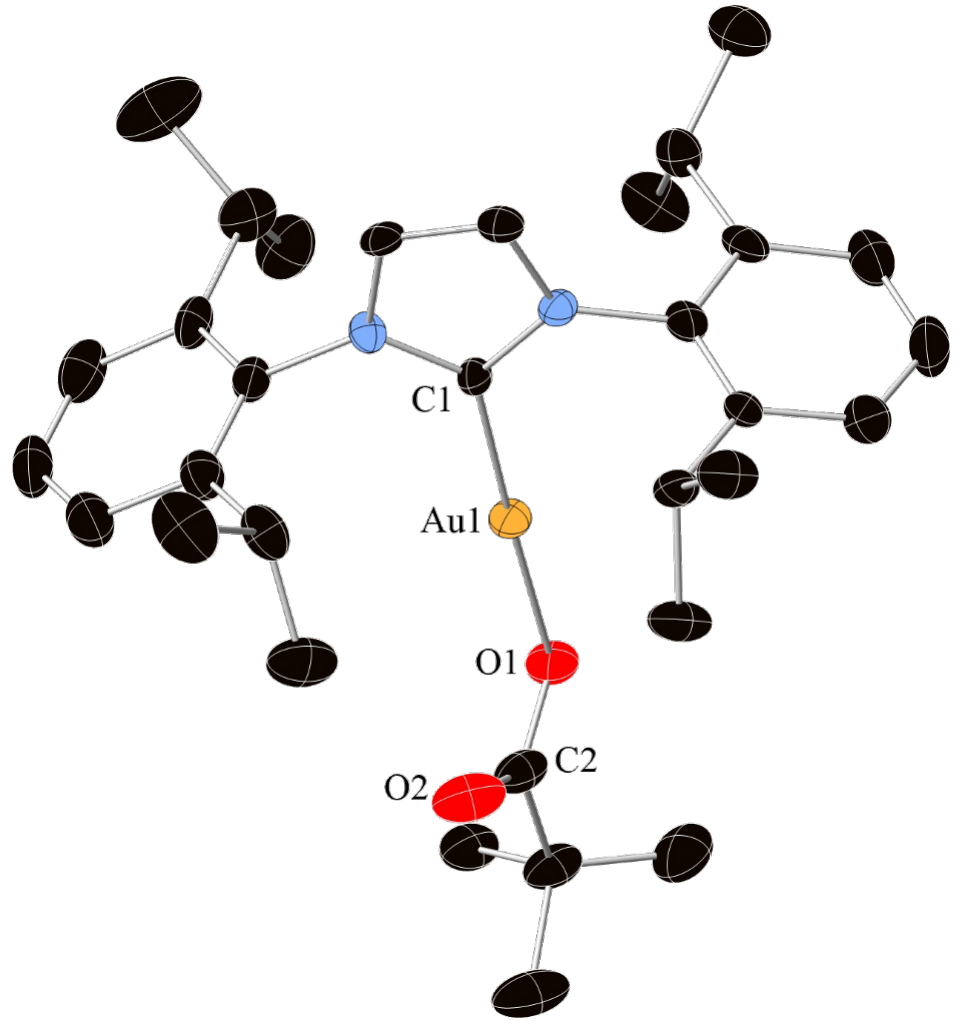
X-ray structure of [Au(OPiv)(IPr)] **3**. Thermal ellipsoids are shown at the 50% probability level. H atoms are omitted for clarity. Selected bond distances (Å) and angles (°) for **3**: Au1–C1 1.978(5), Au1–O1 2.048(4), O1–C2 1.299(8), C2–O2 1.231(9), C1–Au1–O1 178.0(2), Au1–O1–C2 120.7(4), O1–C2–O2 124.6(6).

The reaction between [AuCl(IPr)], **2** and Ag_2_O yielded no product. A stoichiometric addition of pivalic acid was required for the reaction to proceed. The acid was believed to generate the reactive intermediate **3** [Au(OPiv)(IPr)] (OPiv = (CH_3_)_3_CCO_2_) (Figure 1). To verify this hypothesis, the well-defined complex [19] was used in test reactions following the earlier conditions. Complex **3** reacted with **2** in conjunction with Ag_2_O and gave complete conversion to **4** [Au(C_6_H_2_F_3_)(IPr)]. This strongly suggests that complex **3** is indeed an intermediate. However, the use of a silver salt was essential for the reaction to proceed.

The silver salt was suspected of abstracting the halide from [AuCl(IPr)] to generate a possibly active cationic gold(I) species [20]. To test this hypothesis, the well-defined cationic complex [(IPrAu^+^)(NCMe)][BF_4_^-^] [21] was reacted with **2** in the absence of other reagents. No product was observed. Subsequent test reactions were performed using **1** (Table 1, entries 1–3), thus eliminating the possible in situ formation of cationic gold(I). The reaction proved successful in the presence of Ag_2_O, suggesting that silver does not generate cationic gold. The reaction with pivalic acid gave a better conversion, indicating that the gold proceeded via complex **3**. Further reactions used **1** as the gold species and no longer included PivOH.

**Table 1:** C–H functionalisation of **2** using **1**.^a^

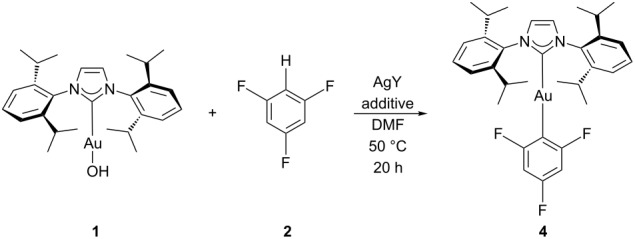			

Entry	AgY	Additive	Conversion^b^ (%)

1	Ag_2_O	-	38
2	Ag_2_O	PivOH	100
3	-	PivOH	0
4^c^	Ag_2_O	-	15
5^d^	Ag_2_O	-	11
6^e^	Ag_2_O	-	0
7	AgF	-	100
8	AgOAc	-	100

^a^Unless otherwise noted, all reactions were carried out with **1** (1 equiv), **2** (4.5 equiv), salt (1.5 equiv) and additive (2.5 equiv) in a 0.2 M DMF solution. ^b^Conversion to product was determined by ^1^H NMR analysis relative to **1**. ^c^AgY (0.5 equiv) was used. ^d^AgY (0.25 equiv) was used. ^e^AgY (0.1 equiv) was used.

The stoichiometric dependence of the silver salt was then examined. Both 0.50 and 0.25 equivalents of Ag_2_O gave poor conversions to the product (entries 4 and 5) and further reducing the loading to 0.1 equivalents stopped the reaction entirely (entry 6).

A range of silver salts was compared in reactions involving **1** and **2**. Both AgF and AgOAc gave full conversion to product (entries 7 and 8), while the nature of the silver counter ion led reactivity to decrease in the order Ag_2_O > AgI > AgO, AgBF_4_, AgCl > Ag_2_CO_3_, AgOCOCF_3_ > AgNO_3_ > AgBr. Electronic effects can explain the general reactivity-decreasing trend on going from AgF to AgBr, but steric factors must also be considered to rationalise the anomalous activity of AgI. The Lewis acidity of the silver salts may contribute to initiate the reaction [22], but unsuccessful reactions using Al_2_O_3_, AlCl_3_, Cu_2_O or ZnBr_2_ proved otherwise. The existence of a transient anion exchange between the gold (AuX) and the silver salt (AgY) may generate the active “AuY” complex. However, this was disproved by the successful reaction using AgCl. This would implicate [AuCl(IPr)] as the possible active species. This has already been discounted since it was shown to be unreactive in the model C–H bond functionalisation reaction.

To complete the examination of possibly important parameters enabling the reaction, the role of solvents was investigated. Solvent screening showed that the reaction could proceed in THF (59% conversion), DMF (38%), toluene (35%), 1,4-dioxane (21%) and poorly in cyclopentyl methyl ether (CPME) (3%). The conversions do not mirror the polarity of the solvents and the silver salts were sparingly soluble in every solvent tested [23].

**Scheme 3:**
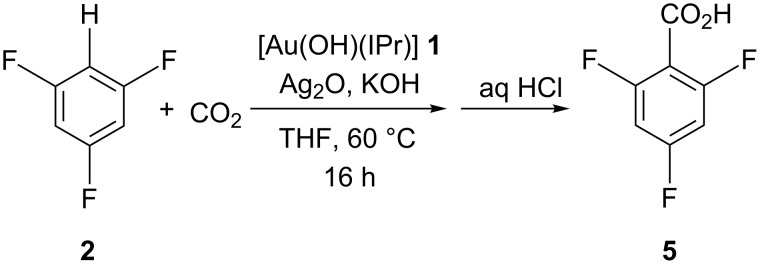
Carboxylation of **2** using **1** and Ag_2_O.

As the functionalisation of C–H bonds was now found possible in the presence of silver additives, we reasoned that a test of the observation was to carry out a carboxylation reaction [12] with substrate **2** using catalyst **1** under optimised carboxylation conditions (Scheme 3).

The general procedure employed **2**, **1** (3 mol %), Ag_2_O (3 mol %) and three equivalents of potassium hydroxide. Gratifyingly, whereas a reaction in the absence of silver leads to no carboxylation product, the addition of silver leads to formation of 2,4,6-trifluorobenzoic acid (**5**). The use of a stoichiometric amount of Ag_2_O results in aggregation of the reagents that seemingly affects mass transport of CO_2_ and halts the reaction. However, the catalytic use of Ag_2_O gave a 22% isolated yield of **5**. This observation clearly shows that silver can have a positive role in the carboxylation of C–H bonds.

## Conclusion

The C–H functionalisation of arenes using (NHC)gold(I) complexes has been shown to be significantly affected by the leaving group on the gold. The gold(I) chloride may only react by first forming the intermediates **1** or **3**. The gold(I) pivalate gives full conversion and is believed to form a six-membered transition state with the substrate. The gold(I) hydroxide gives incomplete conversion to product and the reaction pathway is currently unknown. The use of K_2_CO_3_ was shown to be unnecessary. The solvent used affects the conversion, although polarity appears not to be a factor. Silver does not act as a simple Lewis acid, as shown by the failure of other metal salts to facilitate the reaction. The reaction was shown to require stoichiometric amounts of the silver salt. The failed reaction of a cationic gold species hints that the silver salt has a role other than abstracting halides. The successful reactions of **1** and **3**, which cannot be dissociated by silver salts, reinforce this idea. The choice of silver salt is very important, and has been shown to widely influence the conversion achieved. The high electronegativity of the silver counter ion is of great importance. Finally, evidence of an interaction between silver salts and the substrate suggests that silver activates the aryl C–H bond and is then implicated in a transmetalation reaction with gold to provide the product. The value of silver additives in catalytic carboxylation of C–H bonds was then illustrated in the formation of 2,4,6-trifluorobenzoic acid, which was hitherto unattainable by gold(I)-catalysed carboxylation using catalyst **1** alone. This may provide a method to increase the range of C–H bonds amenable to functionalisation through gold-mediated carboxylation. Studies aimed at examining the extent of this effect are ongoing in our laboratories.

## Supporting Information

File 1 Detailed experimental procedures for the synthesis of complexes **3**–**5**.
