# Supplementary material for: The role of silver additives in gold-mediated C–H functionalisation
Source: Beilstein J Org Chem. 2011 Jul 1;7:892–6. doi: 10.3762/bjoc.7.102 (PMC3135131; doi:10.3762/bjoc.7.102)
Supplement: File 1 — Detailed experimental procedures for the synthesis of complexes 3–5. [file Beilstein_J_Org_Chem-07-892-s001.pdf]

Supporting Information

for

The role of silver additives in gold-mediated C–H functionalisation

Scott R. Patrick, Ine I. F. Boogaerts, Sylvain Gaillard, Alexandra M. Z. Slawin and Steven P. Nolan*

Address: EaStCHEM School of Chemistry, University of St Andrews, North Haugh, St Andrews, KY16 9ST, UK.

Email: Scott R. Patrick - srp3@st-andrews.ac.uk; Ine I. F. Boogaerts - ib51@st-andrews.ac.uk; Sylvain Gaillard - sg210@st-andrews.ac.uk ; Alexandra M. Z. Slawin - amzs@st-andrews.ac.uk; Steven P. Nolan* - snolan@st-andrews.ac.uk

* Corresponding author

Detailed experimental procedures for the synthesis of complexes 3–5

General considerations: All reagents were used as received. ^{1}H, ^{13}C and ^{19}F NMR spectra were recorded on a Bruker Avance 300 or a Bruker Avance II 400 Ultrashield spectrometer. Elemental analyses were performed by the London Metropolitan University Elemental Analysis Service. Complex **1** has been previously described in the literature [1].

Synthesis of [Au(OPiv)(IPr)] (3): A scintillation vial was charged with [Au(OH)(IPr)] (100.0 mg, 0.166 mmol) and pivalic acid (20.3 mg, 0.199 mmol). Toluene (1.5 mL) was then added to the vial and the reaction mixture stirred at room temperature for 21 h. The solvent was evaporated and the residue washed with pentane to give a stoichiometric yield of a white microcrystalline solid. ^{1}H NMR (400 MHz, CD_2Cl_2): δ 7.56 (t, *J* = 8.0 Hz, 2H, C*H* aromatic), 7.36 (d, *J* = 8.0 Hz, 4H, C*H* aromatic), 7.21 (s, 2H, C*H* imidazole), 2.57 (sept, *J* = 7.0 Hz, 4H, C*H*(CH_3)$_2$), 1.35 (d, *J* = 6.9 Hz, 12H, C*H*$_3$), 1.22 (d, *J* = 6.9 Hz, 12H, C*H*$_3$), 0.96 (s, 9H, (C*H*$_3$)$_3$) ppm. ^{13}C NMR (100 MHz, CD_2Cl_2): δ 146.24 (s, 4C), 134.62 (s, 2C), 130.96 (s, 2C), 124.64 (s, 4H), 123.84 (s, 2C), 29.24 (s, 4C), 28.50 (s, 3C), 24.29 (d, *J* = 25.2 Hz, 8C) ppm. Anal. Calcd. for $C_{31}H_{45}AuN_2O$: C, 56.53; H, 6.89; N, 4.25. Found: C, 55.85; H, 6.95; N, 4.11.

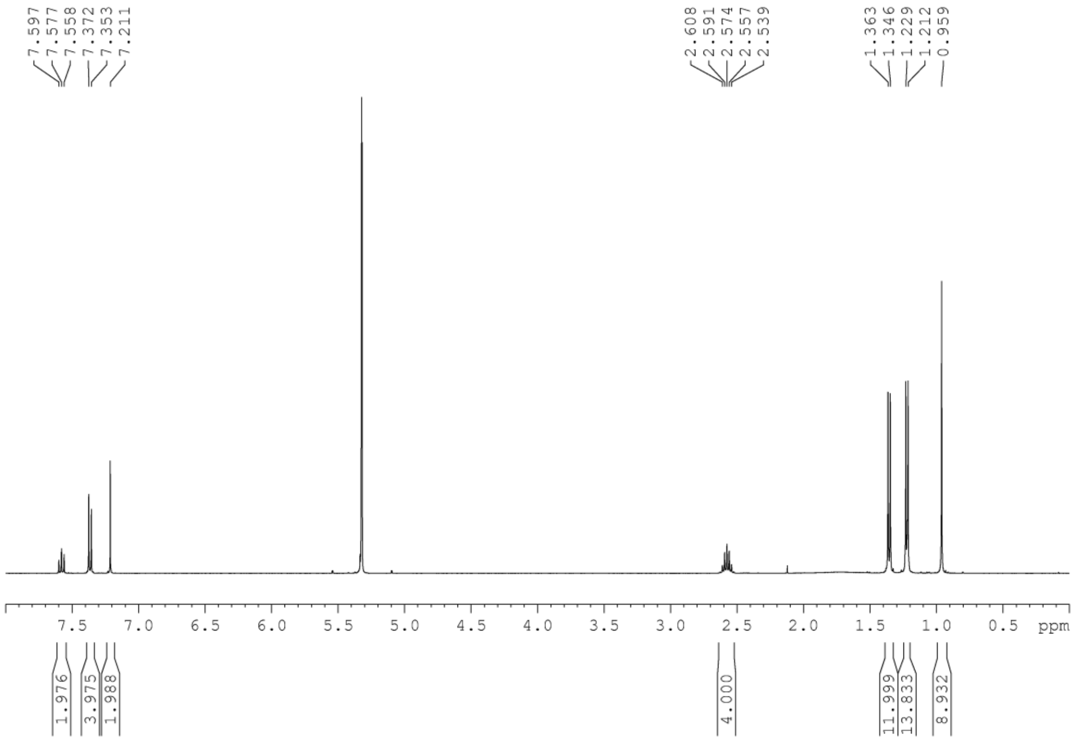

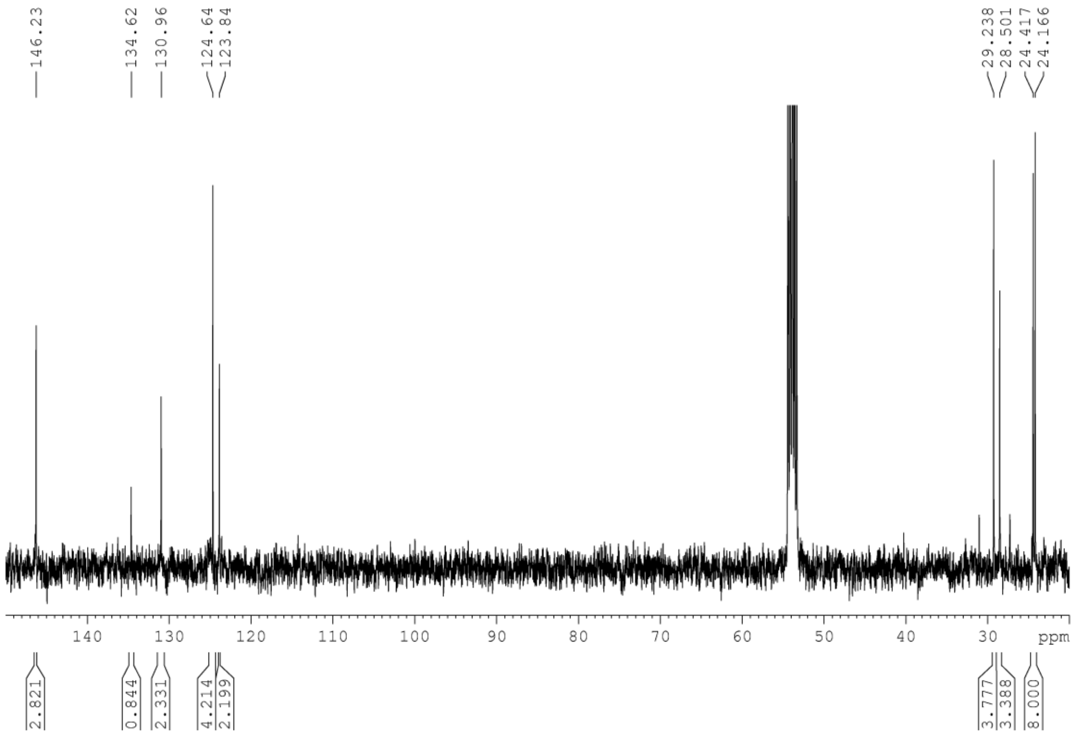

General procedure for synthesis of [Au($C_6H_2F_3$)(IPr)] (4): A scintillation vial was charged with [Au(OH)(IPr)] (25.0 mg, 0.041 mmol), silver salt (0.060 mmol) and 1,3,5-trifluorobenzene (24.4 mg, 19.1 µL, 0.185 mmol). DMF (0.8 mL) was then added to the vial and the reaction mixture stirred at 50 °C for 20 h. The resulting mixture was filtered through Celite® and the solvent evaporated. The residue was purified via flash chromatography (20% DCM/pentane) to afford a pale yellow microcrystalline solid. ^{1}H NMR (400 MHz, CD_2Cl_2): δ 7.50 (t, *J* = 7.8 Hz, 2H, C*H* aromatic), 7.32 (d, *J* = 7.6 Hz, 4H, C*H* aromatic), 7.23 (s, 2H, C*H* imidazole), 6.31 (q, *J* = 4.8 Hz, 2H, C*H* aromatic), 2.64 (sept, *J* = 7.0 Hz, 4H, C*H*(CH_3)$_2$), 1.36 (d, *J* = 6.8 Hz, 12H, CH_3), 1.24 (d, *J* = 6.8 Hz, 12H, CH_3) ppm. ^{13}C NMR (100 MHz, CD_2Cl_2): δ 146.35 (s, 2C), 134.76 (s, 1C), 130.64 (s, 2C), 124.32 (s, 4C), 123.52 (s, 2C), 30.99 (s, 1C), 29.20 (s, 4C), 24.25 (d, *J* = 34.1 Hz, 8C) ppm. ^{19}F NMR (376 MHz, CD_2Cl_2): δ −85.82 (d, *J* = 6.4 Hz, 2F), −117.53 (t, *J* = 6.4 Hz, 1F) ppm. Anal. Calcd. for $C_{33}H_{38}AuN_2F_3$: C, 55.31; H, 5.34; N, 3.91. Found: C, 55.45; H, 5.41; N, 3.83.

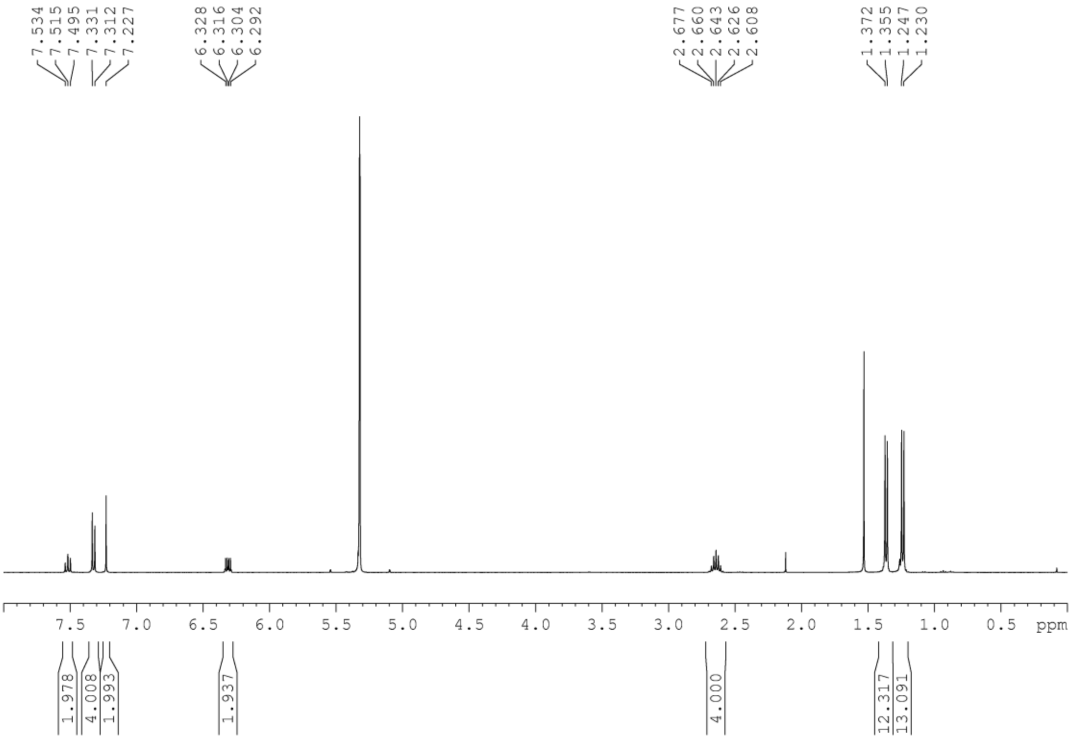
7.534
7.515
7.495
7.331
7.312
7.227
6.328
6.316
6.304
6.292
2.677
2.660
2.643
2.626
2.608
1.372
1.355
1.247
1.230
ppm
1.978
4.008
1.993
1.937
4.000
12.317
13.091

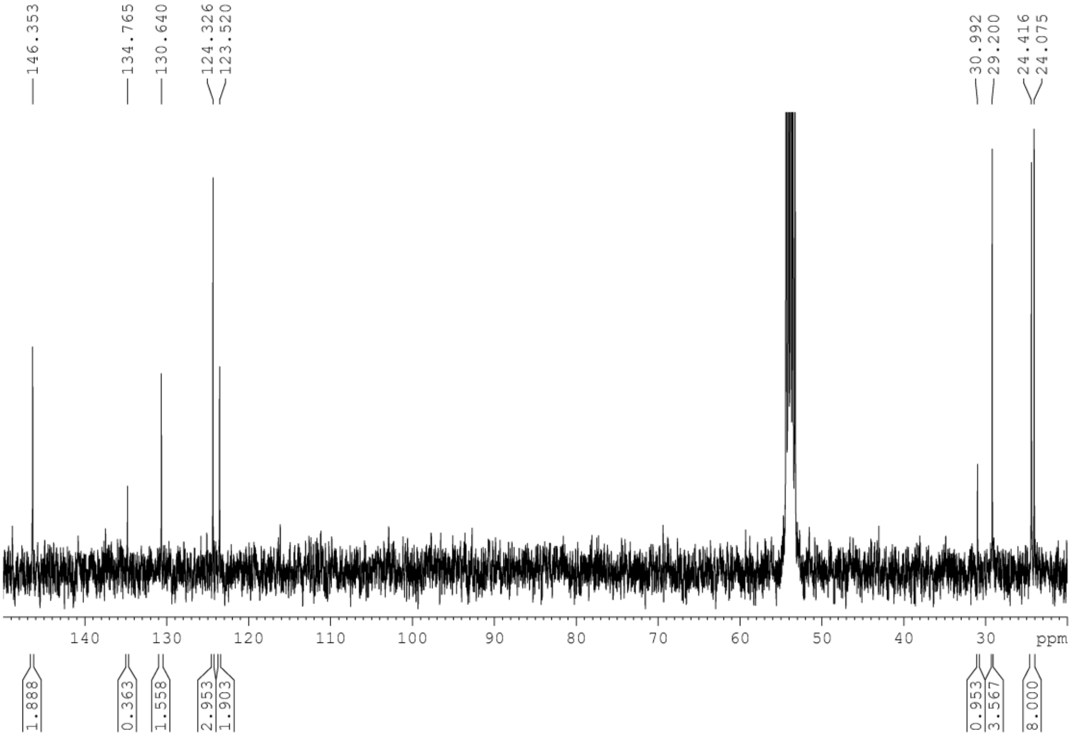
146.353
134.765
130.640
124.326
123.520
30.992
29.200
24.416
24.075
ppm
1.888
0.363
1.558
2.953
1.903
0.953
3.567
8.000

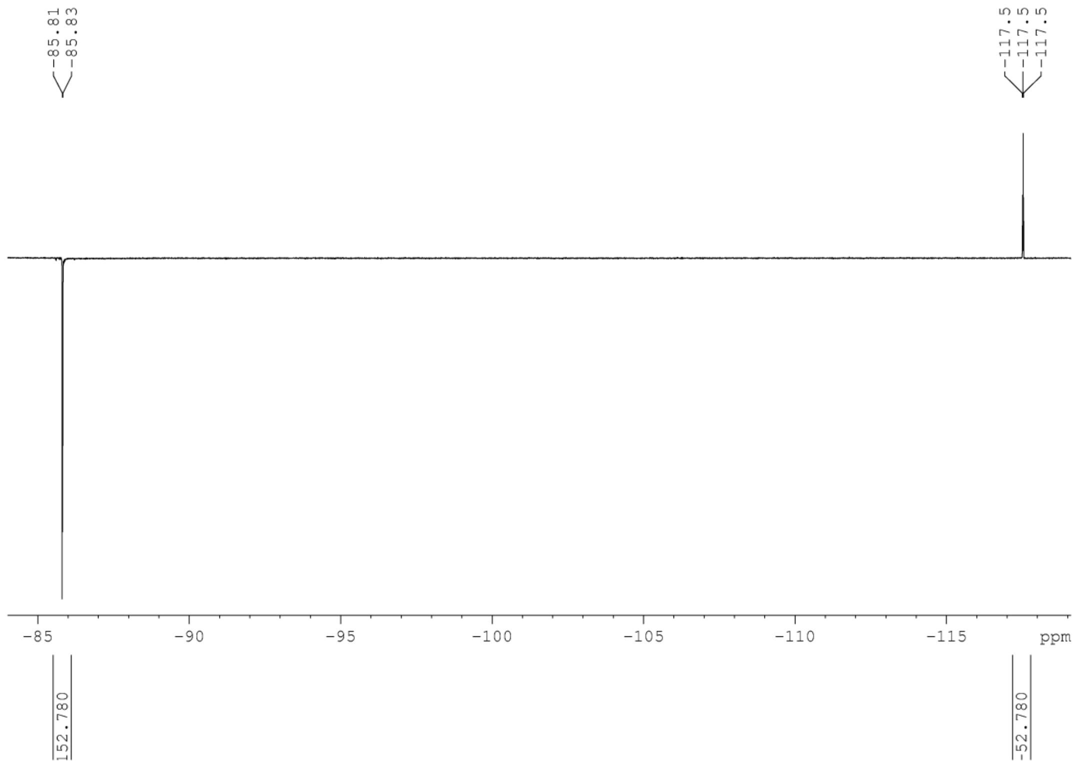

General procedure for synthesis of 2,4,6-trifluorobenzoic acid (5): A pressure tube was charged with 1,3,5-trifluorobenzene (0.5 mmol), [Au(OH)(IPr)] (3 mol %), silver(I) oxide (3 mol %) and potassium hydroxide (1.5 mmol). THF (1.0 mL) was then added to the vial and the tube was placed under a CO_2 atmosphere (2 bar). The reaction mixture was stirred at 60 °C for 16 h and the solvent evaporated. The residue was dissolved in water (2 mL) and filtered through cotton. Aqueous HCl (1M, 0.5 mL) was added to the filtrate and the mixture stirred for 30 minutes. The organic product was then extracted with ethyl acetate (3 × 3 mL). The solvent was evaporated to yield a yellow microcrystalline solid. ^{1}H NMR (300 MHz, CD_2Cl_2): δ 10.31 (br s, 1H), 6.80 (t, J = 8.23 Hz, 2H) ppm. ^{19}F NMR (282 MHz, CD_2Cl_2): δ −100.79 (t, J = 10.2 Hz, 1F), −105.11 (d, J = 10.6 Hz, 2F) ppm.

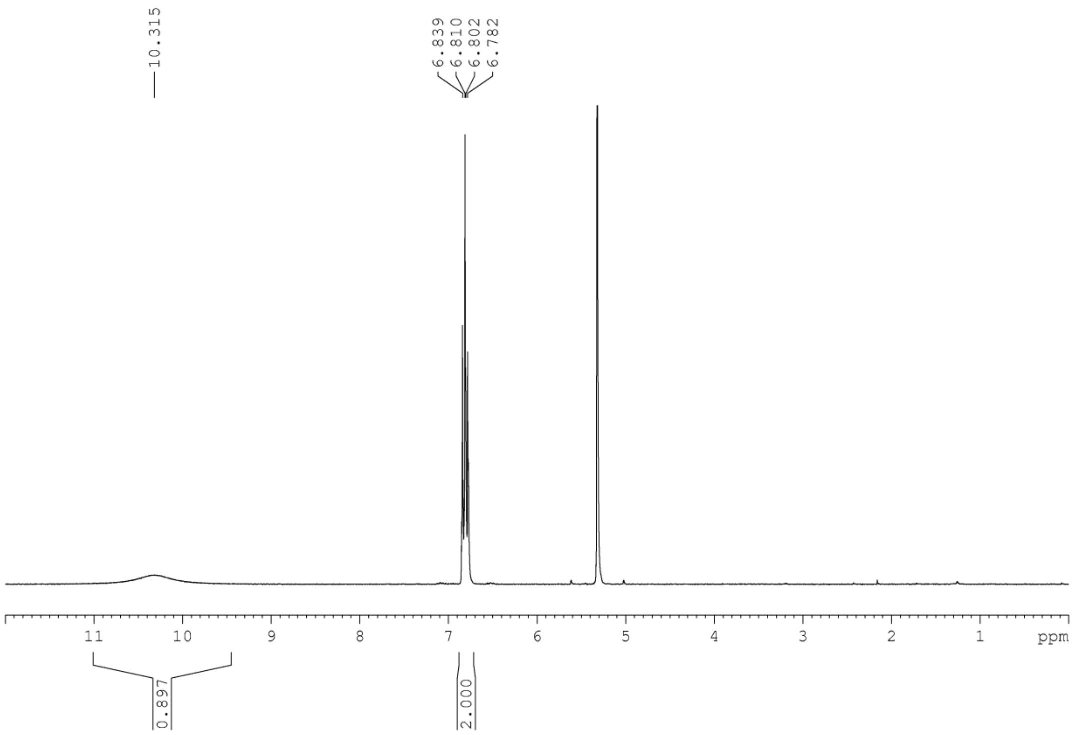
10.315
6.839
6.810
6.802
6.782
11
10
9
8
7
6
5
4
3
2
1
ppm
0.897
2.000

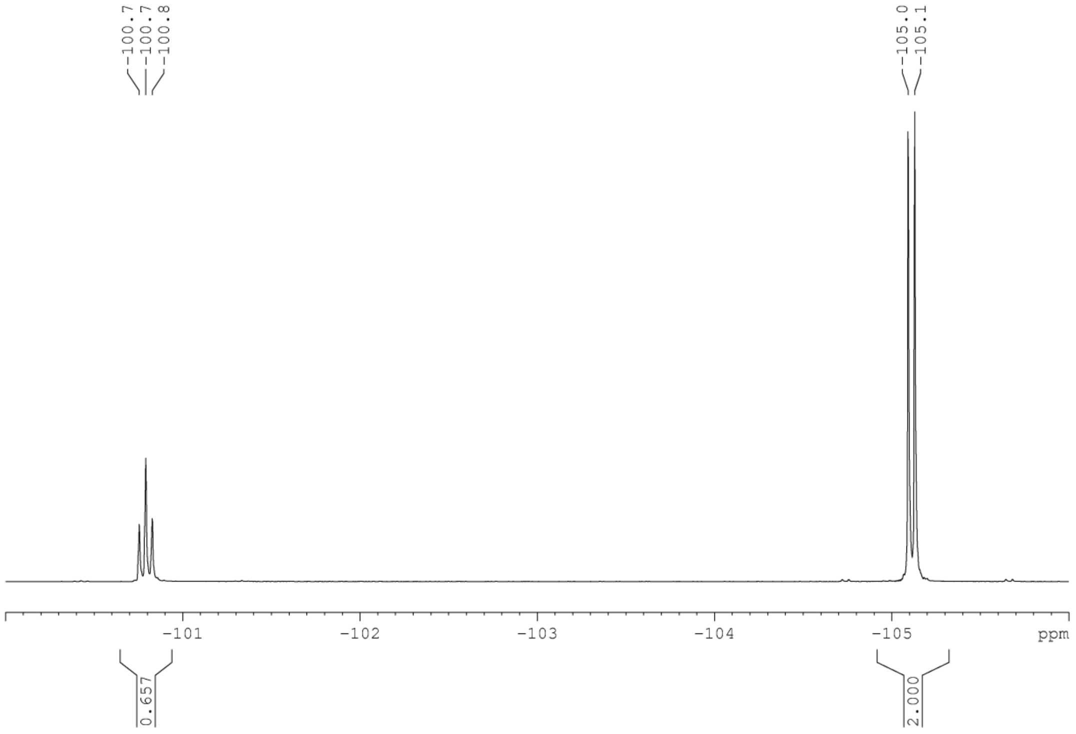
-100.7
-100.7
-100.8
-105.0
-105.1
-101
-102
-103
-104
-105
ppm
0.657
2.000

Conversions using Complex 1[a]:

Entry	Additive(s)	Solvent	Conversion[b] (%)
1	Ag_2O	DMF	38
2	Ag_2O, PivOH	DMF	100
3	PivOH	DMF	0
4[c]	Ag_2O	DMF	15
5[d]	Ag_2O	DMF	11
6[e]	Ag_2O	DMF	0
7	AgF	DMF	100
8	AgOAc	DMF	100
9	AgI	DMF	35
10	$AgBF_4$	DMF	30
11	AgO	DMF	30
12	AgCl	DMF	28
13	Ag_2CO_3	DMF	20
14	$AgOCOCF_3$	DMF	19
15	$AgNO_3$	DMF	13
16	AgBr	DMF	7
17	Al_2O_3	DMF	0
18	$AlCl_3$	THF	0
19	Cu_2O	DMF	0
20	$ZnBr_2$	DMF	0
21	Ag_2O	THF	59
22	Ag_2O	Toluene	35
23	Ag_2O	Dioxane	21
24	Ag_2O	CPME	3

[a]Unless otherwise noted, all reactions were carried out with [Au(OH)(IPr)] (1 equiv), 1,3,5-trifluorobenzene (4.5 equiv), metal salt (1.5 equiv) and pivalic acid (2.5 equiv) in a 0.2M DMF solution. [b]Conversion to product was determined by ^1H NMR analysis relative to [Au(OH)(IPr)]. [c]Metal salt (0.5 equiv) was used. [d]Metal salt (0.25 equiv) was used. [e]Metal salt (0.1 equiv) was used.

NMR spectra of reaction between 1,3,5-trifluorobenzene and Ag_2O:

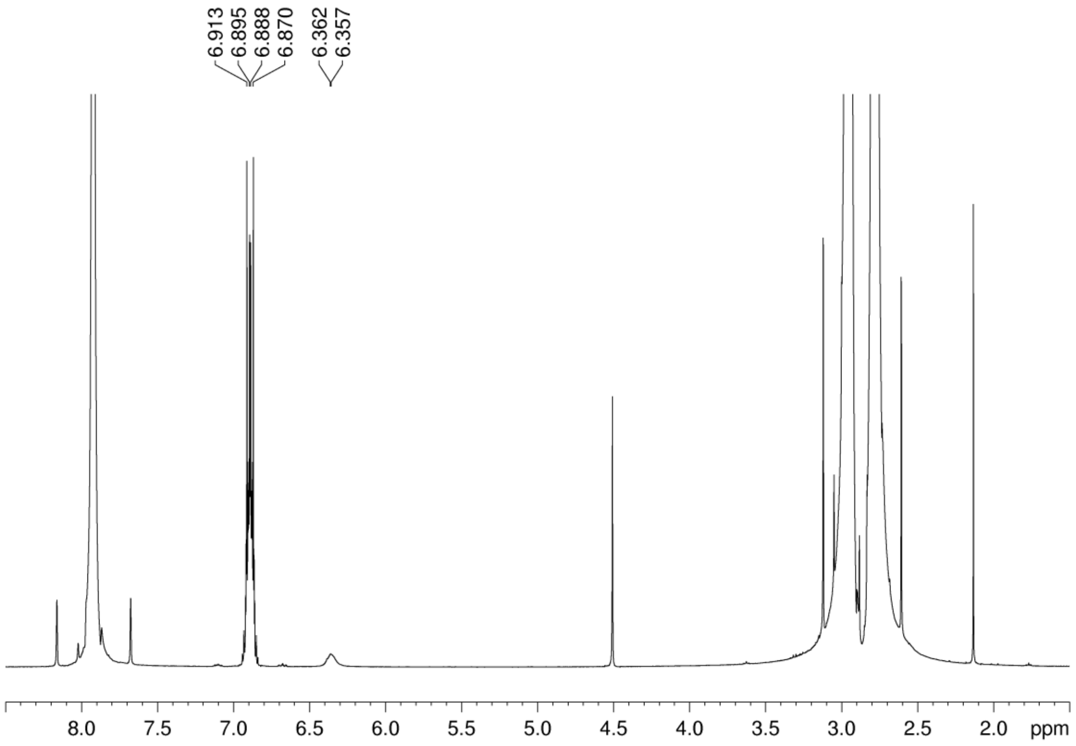

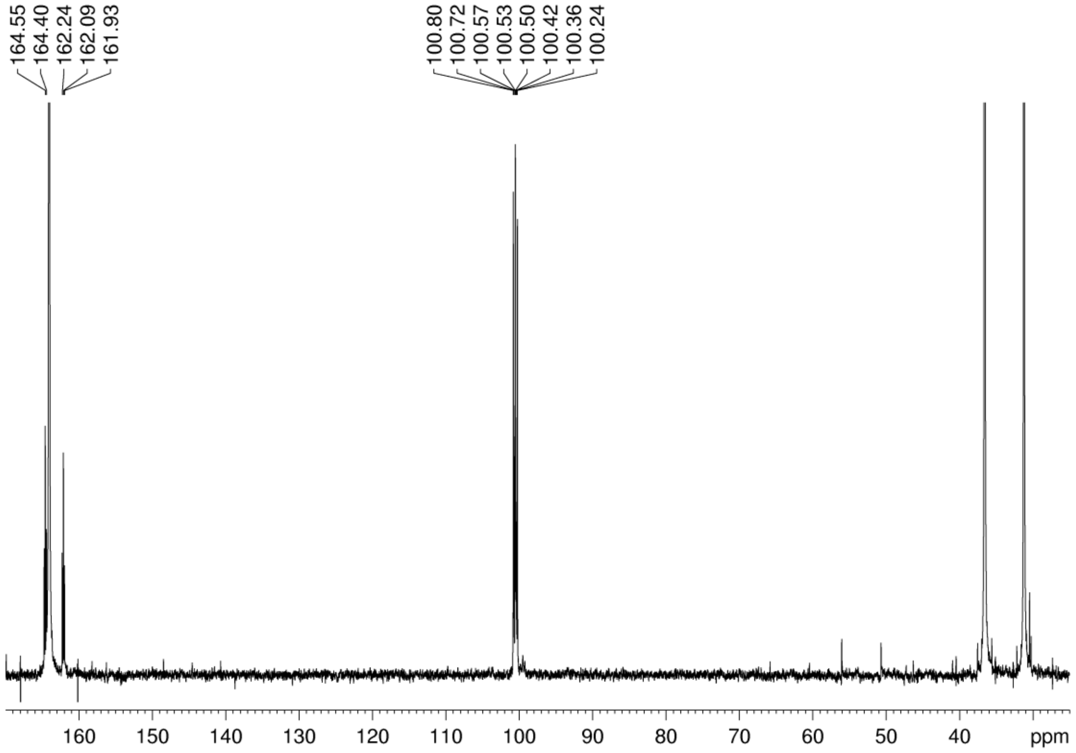

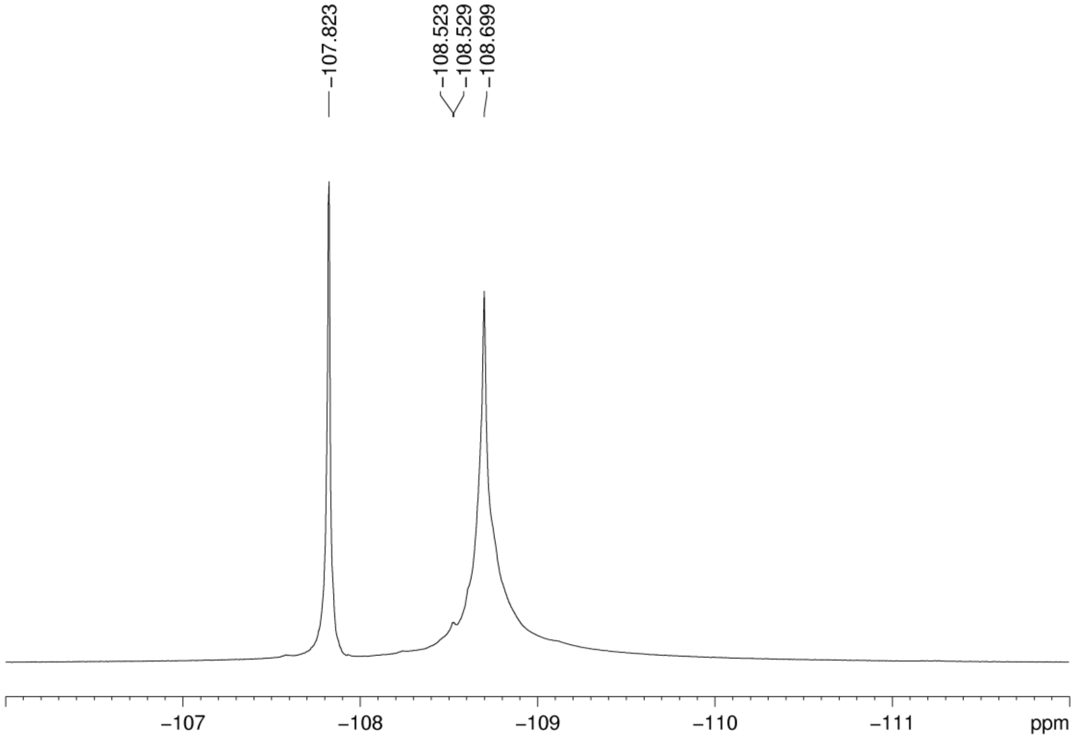

1. Gaillard, S.; Slawin, A. M. Z.; Nolan, S. P. *Chem. Commun.* **2010**, *46*, 2742–2744.
